# Do one-stage indications predict success following two-stage arthroplasty for chronic periprosthetic joint infection?

**DOI:** 10.5194/jbji-9-75-2024

**Published:** 2024-02-23

**Authors:** Michael M. Kheir, Christopher G. Anderson, Yu-Fen Chiu, Alberto V. Carli

**Affiliations:** 1 Department of Orthopaedic Surgery, University of Michigan, Ann Arbor, Michigan, USA; 2 Department of Orthopaedic Surgery, Virginia Commonwealth University, Richmond, Virginia, USA; 3 Department of Orthopedic Surgery, Hospital for Special Surgery, New York, New York, USA

## Abstract

**Introduction**: The 2018 International Consensus Meeting (ICM) proposed criteria for one-stage exchange arthroplasty in treating periprosthetic joint infection (PJI). Our study aimed to determine what proportion of PJI patients met the 2018 ICM criteria and how this affected infection-free survivorship for patients. **Methods**: All chronic PJI patients treated with two-stage exchange within our institution between 2017–2020 were retrospectively reviewed. Included cases met 2011 Musculoskeletal Infection Society (MSIS) criteria for PJI and had a 2-year minimum follow-up. Treatment success was defined as Tier 1A in the 2019 MSIS working group definition. ICM one-stage criteria included non-immunocompromised host, absence of sepsis, adequate soft tissue for closure, known preoperative pathogen, and susceptibility. Immunocompromised host was analyzed as two separate definitions. Kaplan–Meier survivorship, Cox regression, and univariate analyses were performed. **Results**: A total of 293 chronic PJI patients were included. Overall, treatment failure occurred in 64/293 (21.8 %) patients. Only 13 % ( n=37 ) met ICM criteria definition no. 1 for one-stage exchange; 12 % ( n=33 ) met definition no. 2. In both definitions, infection-free survivorship at 2 years did not differ between patients who met and did not meet criteria ( p>0.05 ). Cox proportional hazard regression analyses demonstrated that the only variable predicting treatment failure was knee joint involvement ( p=0.01 ). **Conclusions**: We found that a very limited number of chronic PJI patients were suitable for a one-stage exchange. Furthermore, the supposition that healthier hosts with known pathogens (the basis of the ICM criteria) yield better PJI treatment outcomes was not observed. These results justify the ongoing multicenter randomized control trial comparing one-stage versus two-stage treatment for chronic PJI.

## 1 Introduction

Periprosthetic joint infection (PJI) is a devastating complication that can occur following total joint arthroplasty (TJA). The occurrence of a PJI is estimated to range from 1 %–3 % and can have a detrimental effect on a patient's physical performance (Nguyen et al., 2016; Thiesen et al., 2021). A key aspect of the pathogenesis of PJI is the ability of an invading bacterial or fungal organism to form “biofilm” on the surface of arthroplasty implants. Once formed, biofilm protects pathogens from immune cells, antibiotics, and even mechanical debridement (Shoji and Chen, 2020). Due to the pathophysiology of PJI, the management of these patients requires a thorough surgical debridement, removal, and replacement of implanted components and antibiotic therapy.

To date, the “gold standard” of PJI treatment is the two-stage revision, whereby the infected implant is removed, accompanying tissue is resected, and a temporary cemented construct containing antibiotics is placed (Choi et al., 2013). Despite being known as the most successful surgical intervention for chronic PJI, two-stage revisions are associated with high perioperative complication rates and long hospitalizations (Kildow et al., 2020). Consequently, the “one-stage” revision, where a definitive arthroplasty implant is placed instead of a spacer, has increased in popularity due to its faster functional recovery (Jackson and Schmalzried, 2000) and successful case series outcomes (Gehrke et al., 2013). Despite this enthusiasm, the indications for undertaking a one-stage revision are inconsistent within the literature, with host status, organism virulence, number of previous surgeries, and type of implant used for definitive fixation varying substantially among published studies.

One approach to identify ideal candidates for one-stage revisions is to study two-stage revision patients. If patient- or infection-related factors for successfully carrying out a two-stage procedure are identified, then it would follow logic to assume that these factors could serve as indications for a one-stage revision. Such a study was originally undertaken by Dombrowski et al. (2020), who determined that so-called indications for single-stage revision did confer any improved success rates in patients undergoing two-stage revisions. While innovative in its approach, the indications this study utilized deviated from those derived from the 2018 International Consensus Meeting (ICM). Furthermore, the study sample size was small, the term “immunocompromised state” was not formally defined, and there was no differentiation between hip and knee PJI.

Consequently, the purpose of the current study was to determine if the 2018 ICM indications for one-stage revision confer a protective effect for chronic PJI patients undergoing two-stage revision of the hip and knee. Furthermore, we compared two different definitions of an “immunocompromised state” to determine which one predicted two-stage PJI treatment success.

## 2 Methods

A retrospective review was performed of all patients undergoing removal of a total hip arthroplasty (THA) or total knee arthroplasty (TKA) for treatment of PJI within our high-volume, academic institution from 1 January 2017 to 1 January 2020. Institutional Review Board (IRB) approval was obtained prior to the start of the study. A 2-year minimum follow-up was required for study inclusion or earlier if PJI-related failure had occurred. Included PJI cases at our institution occurred ≥3 months following index surgery and were defined by 2011 Musculoskeletal Infection Society (MSIS) criteria (Parvizi et al., 2011). Specifically, the criteria to declare a diagnosis of PJI are presence of a sinus tract and presence of the same microorganism in two separate culture or fluid samples within the prosthetic joint or, when four of the six criteria were found, elevated erythrocyte sedimentation rate (ESR ≥30 mm h^-1^), C-reactive protein (CRP ≥10 mg L^-1^), elevated synovial neutrophil percentage (PMN% ≥65 %), presence of purulence in the affected joint, isolation of a microorganism in one culture of tissue or fluid, and more than 5 neutrophils per high-powered view on histologic section examination. Systemic host grade was determined by the McPherson classification of periprosthetic infection (McPherson et al., 1999, 2002).

Inclusion criteria were listed as follows: patients greater than 18 years of age who had a preoperative diagnosis of PJI of the hip or knee based on 2011 MSIS, those who underwent implant removal as part of a two-stage exchange arthroplasty, and PJI cases that have a complete 2-year clinical follow-up or failure. Patient excluded were those with less than 2 years of clinical follow-up; patients treated for PJI with insufficient perioperative information to satisfy the 2011 MSIS criteria; and individuals with satisfactory 2011 MSIS criteria who were treated with a debridement, antibiotics, and irrigation (DAIR) procedure. The presence of primary or revision arthroplasty components at the time of implant removal was recorded but not considered as a variable for inclusion or exclusion.

After applying inclusion and exclusion criteria, a total of 293 patients who received a two-stage exchange arthroplasty were included in our final cohort to determine if they met the 2018 ICM one-stage criteria. Criteria for one-stage exchange arthroplasty based on the ICM are non-immunocompromised host, absence of systemic sepsis, minimal bone loss and soft tissue defect allowing for primary closure, and known pathogen and susceptibility preoperatively (Bialecki et al., 2019). The 2018 ICM criterion for an immunocompromised host is quite vague and inevitably has different interpretations and, consequently, may be a subjective criterion. Because of this we sought to create two separate definitions for an immunocompromised host, which were analyzed separately. The first definition was a host meeting McPherson classification grade C (McPherson et al., 1999, 2002). The second definition sought to define these patients more concretely as patients with comorbidities that place them at higher risk of PJI (Eka and Chen, 2015; Enayatollahi et al., 2016; Kuo et al., 2016; Tan et al., 2016; Zuidhof et al., 2019): patients on chemotherapy; patients currently on steroids; diabetics; intravenous drug users; patients on antirheumatic drugs; and patients with immune deficiency (autoimmune disease or HIV/AIDS), chronic hepatitis, or chronic kidney disease.

Treatment success was defined as Tier 3B, 3D, and 3E according to the 2019 MSIS working group definition, which effectively defines success as having no septic revision following the initiation of PJI treatment (Fillingham et al., 2019). Spacer exchange surgery for mechanical complications, such as a fall or fracture, was not considered as treatment failure.

### Statistical analyses

Student's T test was used to compare continuous variables, while a chi-squared test or Fisher's exact test were used for categorical data. Kaplan–Meier survivorship curve analyses were used to identify if one-stage revision criteria for each definition were significantly associated with treatment failure over time, and the 95 % confidence intervals (CI) and logrank test p values were reported. A Cox proportional hazard regression analysis was performed to identify if specific host features are significantly associated with treatment success, after adjusting for whether patients met one-stage criteria, the presence of diabetes mellitus, age, sex, body mass index (BMI), operative time, joint type, McPherson host grade, and Charlson Comorbidity Index (Charlson et al., 1987). An alpha level of 0.05 was used to denote statistical significance. All tests were two-tailed. Statistical analyses were performed using SAS 9.4 (SAS Institute Inc., Cary, NC).

## 3 Results

### 3.1 One-stage criteria with definition no. 1: immunocompromised status based on McPherson grading

Of the 293 patients, only 13 % (37/293) of patients were identified to meet the ICM criteria for one-stage exchange. Baseline demographics for age showed a difference between both patient groups, with those who met the one-stage criteria found to be older (68.9 vs 65.0 years, p=0.03 ). In addition, patients who met ICM criteria had longer hospital stays (11.8 vs 8.9 d, p=0.04 ) but better McPherson host grade distribution ( p=0.002 ) as expected due to the definition used in this group. There was no difference between groups in terms of body mass index (BMI), operative time, sex distribution, joint distribution, proportion of patients with diabetes, ASA (American Society of Anesthesiologists) scores, Charlson Comorbidity Index scores, or presence of primary or revision components (Table 1).

**Table 1:** Demographics of entire cohort and comparison of those that met one-stage criteria (with definition no. 1: immunocompromised based on McPherson grading) with patients that did not.

	All		One-stage indication				
			No		Yes		
			256 (87 %)		37 (13 %)		
	Mean	SD	Mean	SD	Mean	SD	p value
Age (years)	65.5	10.4	65.0	10.5	68.9	9.0	0.03
BMI (kg m^-2^)	31.0	7.2	31.1	7.0	30.7	8.9	0.79
Operative time (minutes)	187.9	66.8	186.5	68.7	198.1	51.7	0.23
Length of stay (days)	9.3	8.0	8.9	7.7	11.8	9.6	0.04
	N	%	N	%	N	%	p value
Sex							0.09
Women	117	39.9	107	41.8	10	27	
Men	176	60.1	149	58.2	27	73	
ASA score							0.42
2	154	52.6	137	53.5	17	45.9	
3	136	46.4	117	45.7	19	51.4	
4	3	1	2	0.8	1	2.7	
Joint							0.88
Hip	130	44.4	114	44.5	16	43.2	
Knee	163	55.6	142	55.5	21	56.8	
McPherson host grade							0.002
A	114	38.9	104	40.6	10	27	
B	142	48.5	115	44.9	27	73	
C	37	12.6	37	14.5	.	0	
CCI							0.11
0	125	42.7	115	44.9	10	27	
1	110	37.5	93	36.3	17	45.9	
3	58	19.8	48	18.8	10	27	
Diabetes							0.70
No	247	84.3	215	84	32	86.5	
Yes	46	15.7	41	16	5	13.5	
Implants							
Primary	145	49.5	127	87.6	18	12.3	0.442
Revision	148	50.5	127	85.8	21	14.2	
Reimplantation							0.20
No	43	15	35	14	8	22	
Yes	250	85	221	86	29	78	

SD: standard deviation. BMI: body mass index. ASA: American Society of Anesthesiologists. CCI: Charlson Comorbidity Index.

**Figure 1:**
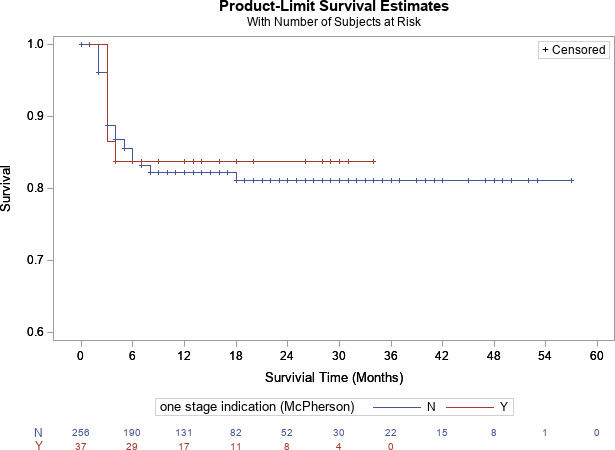
Kaplan–Meier survivorship analysis for infection-free survivorship among all patients who met and did not meet one-stage criteria (definition no. 1).

**Figure 2:**
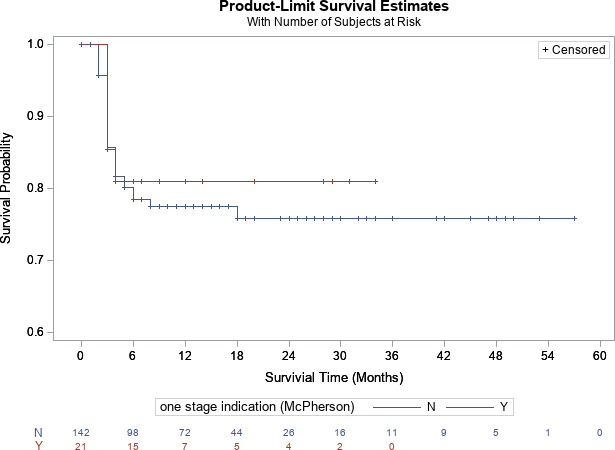
Kaplan–Meier survivorship analysis for infection-free survivorship among knee patients who met and did not meet one-stage criteria (definition no. 1).

**Figure 3:**
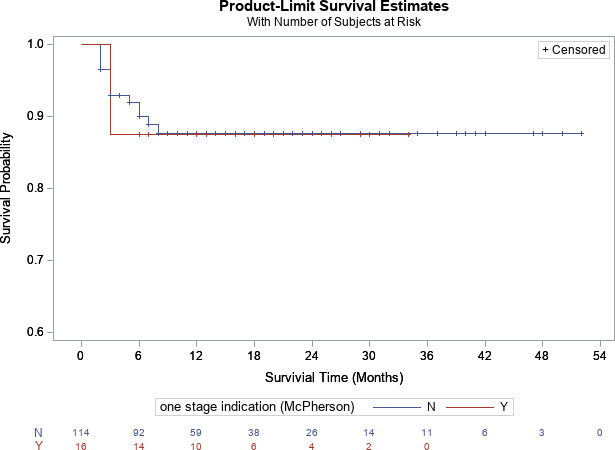
Kaplan–Meier survivorship analysis for infection-free survivorship among hip patients who met and did not meet one-stage criteria (definition no. 1).

Overall, treatment failure occurred in 64/293 (21.8 %) patients. Treatment failure occurred in 8/37 (21.6 %) of patients in the ICM criteria group and 56/256 (21.9 %) of those who did not meet criteria; no statistically significant difference was found between each group ( p=0.97 ). Furthermore, there was no difference in infection-free survivorship between patients who met (83.8 %) [95 % CI = 71.9 %–100 %] and did not meet ICM criteria (81.2 %) [95 % CI = 76.0 %–100 %] at 2-year follow-up, respectively ( p=0.83 , Fig. 1). For knees, there was no difference in survivorship between patients who met (80.9 %) [95 % CI = 64.1 %–100 %] and did not meet criteria (75.8 %) [95 % CI = 68.1 %–100 %] at 2-year follow-up, respectively ( p=0.72 , Fig. 2). For hips, there was no difference in survivorship between patients who met (87.5 %) [95 % CI = 71.3 %–100 %] and did not meet criteria (87.7 %) [95 % CI = 81.0 %–100 %] at 2-year follow-up, respectively ( p=0.93 , Fig. 3).

Cox proportional hazard regression analysis determined that the only statistically significant variable predicting treatment failure was joint type, specifically knees having a higher likelihood of failing (HR 2.48, 95 % CI = 1.24–4.93, p=0.01 ); all other variables including Charlson Comorbidity Index score, diabetes, one-stage indication, age, BMI, operative time, and sex were not associated with a higher failure rate (Table 2).

**Table 2:** Cox proportional hazard regression analysis to determine if specific host features are associated with treatment success.

Parameter		HR	95 % confidence limits		p value
Age	Unit = 10 years	0.88	0.66	1.17	0.37
BMI	Unit = 5 kg m^-2^	0.96	0.79	1.17	0.69
Operative time	Unit = 20 min	1.05	0.96	1.15	0.31
Sex	Men vs women	0.98	0.54	1.77	0.93
Joint	Knee vs hip	2.48	1.24	4.93	0.01
McPherson host	B vs A	0.95	0.50	1.81	0.88
	C vs A	0.73	0.27	1.93	0.52
CCI	1 vs 0	1.21	0.60	2.41	0.60
	3+ vs 0	1.77	0.70	4.44	0.23
Diabetes	Yes vs no	1.07	0.46	2.51	0.87
Met one-stage criteria (definition no. 1: McPherson grading)	Yes vs no	0.85	0.35	2.07	0.71

BMI: body mass index. CCI: Charlson Comorbidity Index. HR: hazard ratio.

### 3.2 One-stage criteria with definition no. 2: immunocompromised status based on comorbidities

Of the 293 patients, only 12 % (33/293) of patients were identified to meet the ICM criteria for one-stage exchange using our immunocompromised definition based on medical comorbidities. Baseline demographics for age showed a difference between both patient groups with those who met the one-stage criteria found to be older (70.3 vs 64.9 years, p=0.004 ). In addition, patients who met ICM criteria had lower BMI (28.4 vs 31.4 kg m^-2^, p=0.03 ). There was no difference between groups in terms of length of hospital stay, operative time, sex distribution, joint distribution, proportion of patients with diabetes, McPherson host grade, ASA scores, Charlson Comorbidity Index scores, or presence of primary or revision components (Table 3).

**Table 3:** Demographics of entire cohort and comparison of those that met one-stage criteria (with definition no. 2: immunocompromised status based on comorbidities) with patients that did not.

	All		One-stage indication				
			No		Yes		
			256 (87 %)		37 (13 %)		
	Mean	SD	Mean	SD	Mean	SD	p value
Age (years)	65.5	10.4	64.9	10.4	70.3	9.4	0.004
BMI (kg m^-2^)	31.0	7.2	31.4	7.3	28.4	6.4	0.03
Operative time (minutes)	187.9	66.8	186.8	68.3	197.3	54.0	0.40
Length of stay (days)	9.3	8.0	8.9	7.1	12.1	13.0	0.18
	N	%	N	%	N	%	p value
Sex							0.95
Women	117	39.9	104	40	13	39.4	
Men	176	60.1	156	60	20	60.6	
ASA score							0.70
2	154	52.6	138	53.1	16	48.5	
3	136	46.4	119	45.8	17	51.5	
4	3	1	3	1.2	–	0	
Joint							0.61
Hip	130	44.4	114	43.8	16	48.5	
Knee	163	55.6	146	56.2	17	51.5	
McPherson host grade							0.43
A	114	38.9	104	40	10	30.3	
B	142	48.5	125	48.1	17	51.5	
C	37	12.6	31	11.9	6	18.2	
CCI							0.19
0	125	42.7	115	44.2	10	30.3	
1	110	37.5	93	35.8	17	51.5	
3	58	19.8	52	20	6	18.2	
Diabetes							0.11
No	247	84.3	216	83.1	31	93.9	
Yes	46	15.7	44	16.9	2	6.1	
Implants							
Primary	145	49.5	129	88.9	16	11.1	0.412
Revision	148	50.5	129	87.2	19	12.8	
Reimplantation							0.26
No	43	15	36	14	7	21	
Yes	250	85	224	86	26	79	

SD: standard deviation. BMI: body mass index. ASA: American Society of Anesthesiologists. CCI: Charlson Comorbidity Index.

**Figure 4:**
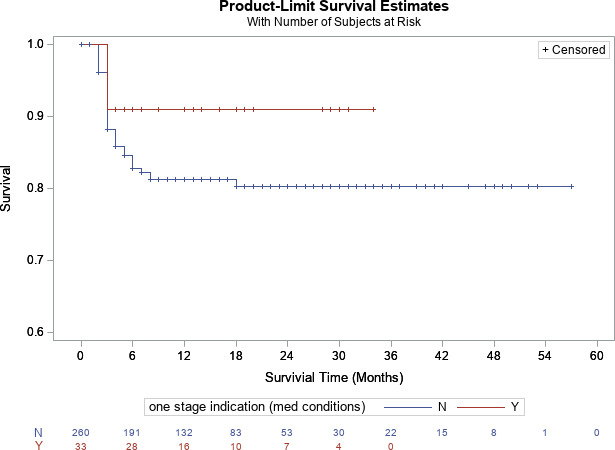
Kaplan–Meier survivorship analysis for infection-free survivorship among all patients who met and did not meet one-stage criteria (definition no. 2).

**Figure 5:**
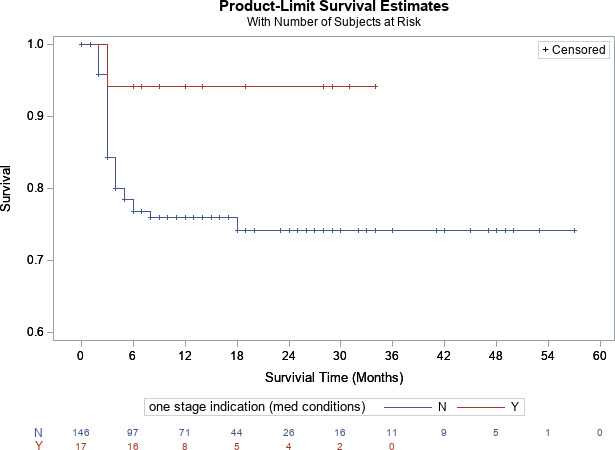
Kaplan–Meier survivorship analysis for infection-free survivorship among knee patients who met and did not meet one-stage criteria (definition no. 2).

**Figure 6:**
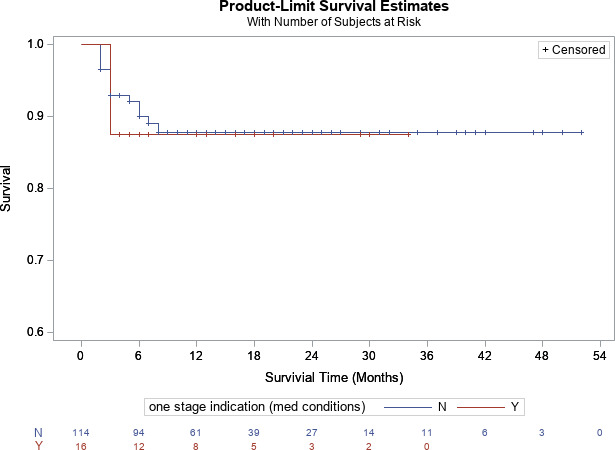
Kaplan–Meier survivorship analysis for infection-free survivorship among hip patients who met and did not meet one-stage criteria (definition no. 2).

Overall, treatment failure occurred in 64/293 (21.8 %) patients. Treatment failure occurred in 6/33 (18.2 %) of patients in the ICM criteria group and 58/260 (22.3 %) of those who did not meet criteria; no statistically significant difference was found between each group ( p=0.589 ). Furthermore, there was no difference in infection-free survivorship between patients who met (90.9 %) [95 % CI = 81.1 %–100 %] and did not meet ICM criteria (80.3 %) [95 % CI = 75.1 %–100 %] at 2-year follow-up, respectively ( p=0.19 , Fig. 4). For knees, there was a trend toward significant difference between patients who met (94.1 %) [95 % CI = 82.9 %–100 %] and did not meet criteria (74.2 %) [95 % CI = 66.5 %–100 %] at 2-year follow-up, respectively ( p=0.10 , Fig. 5). For hips, there was no difference in survivorship between patients who met (87.5 %) [95 % CI = 71.3 %–100 %] and did not meet criteria (87.8 %) [95 % CI = 81.5 %–100 %] at 2-year follow-up, respectively ( p=0.87 , Fig. 6).

Cox proportional hazard regression analysis determined that the only statistically significant variable predicting treatment failure was joint type, specifically knees having a higher likelihood of failing (HR 2.48, 95 % CI = 1.24–4.93, p=0.01 ); all other variables including Charlson score, diabetes, one-stage indication, age, BMI, operative time, and sex were not associated with a higher failure rate (Table 4).

**Table 4:** Cox proportional hazard regression analysis to determine if specific host features are associated with treatment success.

Parameter		HR	95 % confidence limits		p value
Age	Unit = 10 years	0.89	0.68	1.18	0.44
BMI	Unit = 5 kg m^-2^	0.95	0.78	1.16	0.60
Operative time	Unit = 20 min	1.05	0.96	1.15	0.29
Sex	Men vs women	0.97	0.53	1.75	0.91
Joint	Knee vs hip	2.48	1.24	4.93	0.01
McPherson host	B vs A	0.94	0.49	1.80	0.85
	C vs A	0.77	0.29	2.03	0.59
CCI	1 vs 0	1.24	0.62	2.47	0.55
	3+ vs 0	1.78	0.71	4.44	0.22
Diabetes	Yes vs no	1.05	0.45	2.43	0.92
Met one-stage criteria (definition no. 2: comorbidities)	Yes vs no	0.49	0.15	1.60	0.24

BMI: body mass index. CCI: Charlson Comorbidity Index. HR: hazard ratio.

## 4 Discussion

Given the rise in the number of TJA performed due to the prevalence of the aging population, an increase in the number of surgical complications such as infection and surgical revisions is expected to occur (Choi et al., 2013). With PJI known as one of the most common complications after a TJA, surgical decision-making to determine whether a one-stage or two-stage exchange arthroplasty is needed is paramount. Two-stage exchange arthroplasty has been the gold standard in the surgical treatment of a chronic infection after TKA and THA. However, there is no clear evidence in current literature that a two-stage exchange has a higher success rate than a one-stage procedure. There are significant pre, peri-, and post-operative details that have been described and have been historically respected in order to fulfill a one-stage surgical approach (Gehrke et al., 2013; Gulhane et al., 2012; Leonard et al., 2014). These variables as well as shared decision-making can aid both the surgeon and patient to come to an agreement to determine whether a one-stage or two-stage approach should be pursued.

At our institution, patients with chronic PJI are treated almost exclusively with two-stage exchange arthroplasty. Our aims for this study were to (1) determine the number of patients with chronic PJI at our high-volume institution who fulfill the ICM criteria for single-stage revision and (2) determine the percentage of patients who failed treatment that met single-stage criteria versus those who did not. The importance of this question is to adjudicate whether the ICM criteria are helpful in predicting a lower rate of persistent or recurrent infection for those who meet the criteria; the presumption is that the ICM criteria act as a surrogate for determining a healthier host. We found that only a small number of chronic PJI patients (12 %–13 %) who were originally treated via two-stage revision met the ICM criteria for single-stage revision. Furthermore, our results showed that the proposed criteria for performing a one-stage surgery were non-inferior for predicting treatment failure among patients undergoing two-stage surgery during a minimum of 2-year follow-up, regardless of our definition for immunocompromised patients and even when stratified by joint type.

Although our sample size is much larger, our overall findings correlate well with a previous study by Dombrowski et al. (2020), who sought to determine the percentage of patients who met the single-stage criteria and if a clinical difference was noted between those who met the ICM criteria and those who did not. They also found that a low number of patients ( 20/108 , 19 %) met the criteria for single-stage exchange. In addition, the authors did not find a difference in risk of persistent of persistent infection or reinfection at 2-year follow-up between the two groups (20 % vs 32 %, p=0.38 ); with our larger sample size, we found that the difference in treatment failure is much smaller between both groups than previously reported by their group. Prior systematic reviews and small studies have also failed to show a difference in reinfection risk between both cohorts (Kildow et al., 2020; Leonard et al., 2014; Nagra et al., 2016; van den Kieboom et al., 2021).

The suggested advantages of a one-stage procedure include lower mortality and morbidity, lower overall healthcare costs, and improved patient-reported outcomes compared to a two-stage exchange in few studies (Choi et al., 2013; Haddad et al., 2015; Klemt et al., 2021; Lum et al., 2020; Rowan et al., 2018; Yaghmour et al., 2019). However, there remains mixed results regarding these outcomes (Baker et al., 2013; Choi et al., 2013; Klouche et al., 2012). Furthermore, several suggested advantages remain unclear in the literature due to a lack of direct head-to-head comparisons for mortality and morbidity, operative times, risk of arthrofibrosis, among others. Direct comparisons between the two groups are difficult to make because patients that meet one-stage criteria are ultimately a very dissimilar patient population with a different risk profile. However, the data in our study demonstrated no difference in the risk of reinfection between both cohorts. We had expected to see a lower reinfection rate in the one-stage group given their healthier profile. It is possible that had these patients actually undergone a one-stage procedure, we may have seen a higher reinfection rate. This emphasizes the need for a randomized control trial to truly determine superiority between both surgical procedures.

Our study has several limitations, and our findings should be viewed in light of these. Despite being performed in a single institution, debridement of soft tissue and bone is most likely heterogeneous among surgeons, and this could affect treatment success rates. This was a retrospective study which harbors inherent bias due to study design. Treatment success (or failure) has been defined using the recent tiered definitions reported by the MSIS working group to only include patients who underwent surgical reoperation for PJI, and we did not consider other definitions for failure for the purpose of this study. The 2018 ICM criteria for one-stage indications are vague, which leads to inconsistent definitions across studies, including this one. This mostly pertains to the definition of an immunocompromised host, which we attempted to explore using two different definitions: (1) McPherson grading and (2) specific medical comorbidities for an immunocompromised host as mentioned in our methodology. However, this points to a specific issue in the literature in that an “immunocompromised” host is poorly defined in the current ICM criteria. Lastly, although our study is much larger compared to the recent publication by Dombrowski et al (2020), we still remain underpowered to determine any statistical difference. It is possible that a subset of patients may benefit from a one-stage exchange; however, we may have not accounted for important variables that affect infection risk.

In conclusion, we found that a very limited number of chronic PJI patients were suitable for a single-stage exchange. Furthermore, the supposition that healthier hosts with known pathogens (the basis of the ICM criteria) yield better PJI treatment outcomes was not observed. These results justify the ongoing multicenter randomized control trial comparing single-stage and two-stage treatment for chronic PJI.

## Data Availability

Data supporting this study are available from the Stavros Niarchos Complex Joint Reconstruction Center Registry at the Hospital for Special Surgery (HSS). Requests for access to the data are subject to review and approval of HSS and may require a data-sharing agreement to address privacy, confidentiality, and ethical concerns.
